# Ultrasound Pulse-Echo Coupled with a Tracking Technique for Simultaneous Measurement of Multiple Bubbles

**DOI:** 10.3390/s18051327

**Published:** 2018-04-25

**Authors:** Antonin Povolny, Hiroshige Kikura, Tomonori Ihara

**Affiliations:** 1 Laboratory for Advanced Nuclear Energy, Tokyo Institute of Technology, Ookayama, Meguro-ku, Tokyo 152-8550, Japan; kikura@lane.iir.titech.ac.jp; 2 Department of Marine Electronics and Mechanical Engineering, Tokyo University of Marine Science and Technology, Etchujima, Tokyo 135-8533, Japan; ihara@kaiyodai.ac.jp

**Keywords:** ultrasound, two-phase flow, bubble, measurement technique, tracking

## Abstract

Bubbly flows are commonly used in various applications and their measurement is an important research topic. The ultrasound pulse-echo technique allows for the detection of each bubble and the measurement of the position of its surface. However, so far it has been used only to measure single bubbles. This paper investigates whether the pulse-echo technique can be applied for measuring multiple bubbles concurrently. The ultrasonic transducer wavelength and diameter were selected based on expected bubble diameters so that each bubble produced a strong reflection. The pulse-echo was implemented to obtain good accuracy without sacrificing the signal processing speed. A tracking technique was developed for the purpose of connecting detected reflections to trajectories. The technique was tested experimentally by measuring the horizontal position of rising air bubbles in a water tank. The results show that the pulse-echo technique can detect multiple bubbles concurrently. The pulse-echo technique detected almost the same number of bubbles as a high-speed video. For average void fractions up to around 1% (and instantaneous void fraction reaching 5.3%), the rate of bubbles missed by the pulse-echo and the rate of noise trajectories both stayed less than 5%. The error rate increased with the void fraction, limiting the technique’s application range.

## 1. Introduction

Various industrial applications require the accurate prediction of bubbly flows, which are typically described using some averaged parameters (e.g., superficial velocities in a pipe flow). However, prediction based on the averaged parameters is limited to simple flow geometries and conditions. Overcoming this limit requires going beyond the average parameters, in other words observing each individual bubble, understanding its dynamics and the mechanisms of bubble interaction. Such an observation requires a measurement technique with the ability to resolve each bubble individually. Some intrusive techniques, such as optical probe [1] , resistivity probe [1], or wire mesh tomography [2] have this ability. From non-intrusive techniques, a high-speed camera (HSC) with particle tracking velocimetry (PTV) [3] is often used because of its great resolution in both time and space. However, HSC requires an optical access to a measurement volume, which is not always possible such as in the case of optically opaque pipes or fluids. Optical access is not required by electrical capacitance or conductance sensors [4,5,6] nor by the gamma-ray tomography [7,8,9]. However, their resolution does not allow for resolving each bubble in a bubbly flow. Compared to that, an ultrasound, which also does not require optical access, can usually achieve good resolution (both temporal and spatial), and that makes it an attractive option.

Ultrasound measurement techniques apply an ultrasonic transducer (TDX) with a piezoelectric element to generate ultrasonic waves from an electric signal (emit) and vice versa (receive). Ultrasound measurement techniques for bubbly flows can be classified into three groups: transmission techniques, Doppler techniques, and pulse-echo techniques. Transmission techniques measure average void fraction from attenuation and delay of ultrasonic pulses caused by bubbles [10] and Doppler techniques measure velocity profiles [11,12]. While transmission and Doppler techniques measure averaged parameters, i.e., void fraction and velocity, the pulse-echo technique measures the axial (along an ultrasonic beam) position of bubble surfaces based on transit time, i.e., the time required for a pulse to travel to the bubble surface and back. Doppler and pulse-echo techniques use the same physical principle. However, unlike the Doppler technique, the pulse-echo technique must distinguish between reflections from various bubbles and as such is suitable only for flows with large strongly reflecting bubbles and low bubble number densities. In this paper, we focus on the diameter of few millimetres (typical for air bubbles produced by a nozzle or a multi-nozzle injection into water) and average bubble number densities up to $0.6\times 10^{6}$ m${}^{-3}$ (void fraction 1%).

While common in the medical, non-destructive testing, and other fields, the pulse-echo has not been applied often to bubble measurement. The pulse-echo technique was used in [13,14] to measure the radius of a single vapour bubble growing rapidly on a heated surface. A digital signal processing system was used in [15] to measure the bubbling frequency at a nozzle and classify flow regimes in the horizontal pipe two-phase flow [16]. The void fraction and the interfacial area were obtained by assuming the shape of the bubble. The ultrasonic transit time technique [17,18] employed an array TDX to measure rising bubbles at multiple vertical levels. The single bubble regime (or bubble chains regarded as single bubbles) was used and individual bubbles were distinguished from each other using time gaps with no bubble detected. In [17,18], it has been shown that by measuring the position of a bubble surface from different directions or at different vertical levels, the bubble diameter and the vertical (perpendicular to the ultrasonic beam) velocity can be measured. This illustrates the merit of measuring the bubble surface position, which allows for obtaining such additional data.

In the above-mentioned studies, the pulse-echo technique was limited to measuring a single bubble surface at a time. The reason for this is that in many configurations, the bubble closest to the TDX is the only one that can be detected accurately. This was explained in [19], where the pulse-echo technique was applied to a bubbly regime. The position of the closest bubble was measured at each pulse repetition and the time-averaged void fraction was estimated using a probabilistic approach.

In this paper, we hypothesise that the pulse-echo technique is capable of detecting multiple bubbles simultaneously. In other words, even bubbles which are not the closest to the TDX can be detected and positions of their surfaces can be measured with a reasonable accuracy. In order to do so, it is necessary to apply a TDX with a suitable diameter and wavelength of the ultrasound beam, the selection of which is explained in this paper. Moreover, this paper explains the signal processing developed for the purpose of this measurement. The key point of our signal processing is the tracking technique, which distinguishes between different bubbles using the measurement time (number of the ultrasound pulse emissions), position (transit time), and the reflection strength (related to amplitude). In contrast with that, previous reports have applied only the measurement time, which was enough for measuring single bubbles.

The aim of this paper is to confirm our hypothesis experimentally by comparing the results of our technique with a reference measurement by an HSC. The technique was tested using a horizontal measurement of bubble vertex positions for various bubble number densities and constant bubble diameters. Testing the technique under varying bubble diameters, TDX wavelengths, and TDX diameters is beyond the scope of this paper. The authors have been developing this technique and have presented it in [20,21,22,23]. This paper describes in detail the developed technique and its performance under the presence of multiple bubbles in the measurement volume.

## 2. Methods

In the pulse-echo technique, ultrasonic pulses (*n* periods of a basic frequency $f_{0}$) are emitted by a TDX in repetitions (with a pulse repetition frequency $f_{PRF}$). A pulse reflects from bubbles and the TDX records a waveform with all the reflections. Each reflection is delayed behind the pulse-generating signal by a transit time *T*, which relates to the distance *x* between the bubble and the TDX as:

(1) $$x=\frac{1}{2}cT$$

using a sound velocity *c*. The pulse-echo technique measures the transit time of reflections and thus the positions of bubble surfaces. According to [15], on the bubble surface, a small part that is the closest to the TDX and almost parallel to the TDX surface contributes the most to the reflection which gets back to the TDX. We assume that bubble shapes are not too distorted and thus the reflection transit time *T* corresponds to the position of the bubble vertex, the surface point closest to the TDX.

**Figure 1:**
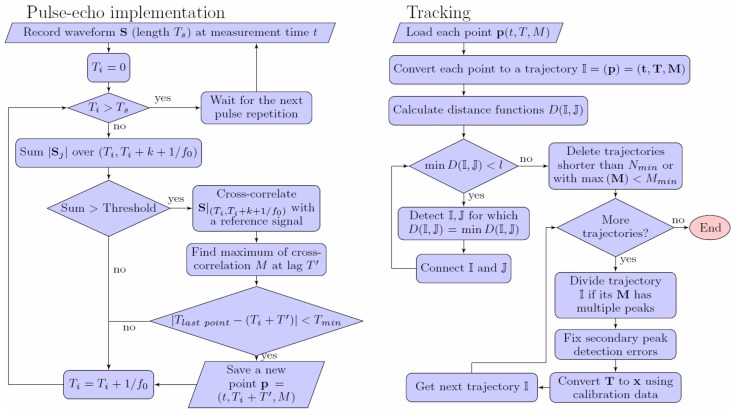
The flow chart of the technique.

Before describing the measurement technique, it is necessary to consider the selection of desired TDX parameters, which allow strong bubble reflections and ensure that the ultrasound is not completely reflected by the closest bubble and can propagate behind it. The technique flowchart is shown in Figure 1. The technique consists of two parts. The first part is a pulse-echo technique, which detects bubble reflections and their transit times in each pulse repetition. The second part is a tracking technique, which connects detected reflections into bubble trajectories.

### 2.1. Transducer Parameter Selection

The pulse-echo technique requires that the ultrasonic pulse reaches the surface of each bubble and reflects back to the TDX with an amplitude above the noise level. These conditions, trivially met in the case of a single bubble, are not always met in the case of multiple bubbles. Because of that, there are occasional measurement errors. A proper selection of TDX parameters is the key to keeping the error rate at a reasonably low level.

First, the TDX diameter was selected so that

(2) D>d

where *D* is the ultrasound beam diameter and *d* is the expected bubble diameter. This condition allows the sound beam to propagate around the bubble. Behind the bubble, there is a shadow cast by the bubble. Nevertheless, a diffraction pattern appears with high sound intensity regions (e.g., Poisson spot) in the shadow. The exact description of how TDX parameters affect the diffraction and the ability of bubbles to reflect from the diffraction zone is a topic that is worth its own paper and should be examined in more detail so that the optimal TDX can be chosen for the pulse-echo. However, in this paper, we focused only on the experimental proof of our hypothesis. Regarding the diffraction, we assumed that unless the wavelength λ is small compared to the bubble diameter, specifically

(3) $$\frac{d}{\lambda }<10,$$

then the ultrasound can propagate behind the obstructing bubble and detect other bubbles there in most cases. It should be noted that the probability of failing to detect a bubble due to obstruction/shadow from another bubble depends also on the bubble number density: the more bubbles, the more obstruction there is, and the greater the number of detection failures.

The reflection strength depends on the type of ultrasound reflection, which changes with the ratio of wavelength and bubble diameter as described in [24]. In order to obtain a high signal-to-noise ratio, total reflection type was applied in this work. The requirement to achieve the total reflection is

(4) $$\frac{d}{\lambda }>1$$

which was used together with (3) for selecting an appropriate TDX frequency $f_{0}$ so that

(5) $$10>\frac{d}{\lambda }>1.$$

### 2.2. Pulse-Echo Technique Implementation

Each waveform was analysed to detect reflections from bubbles and to measure the positions of bubble vertices. The positions were calculated using (1) from transit times, which should be measured as precisely as possible. From various available methods [25] we employed the thresholding method (simple, fast but inaccurate) together with the cross-correlation method (accurate but slow). The thresholding detected bubbles and roughly estimated their transit times. Then, the cross-correlation with a reference signal was used only on a small part of the waveform (to decrease the computation time) and improved the estimation of the transit time. The reference signal was obtained by measuring the reflection from a free water surface. The length of the reference signal *k* was chosen to include all the peaks higher than 5% of the highest peak in the reference signal.

#### 2.2.1. Thresholding Method

The implementation of the thresholding followed the typical scheme described in [25] with a few minor changes. To make the algorithm simpler and slightly faster, the algorithm used the sum of absolute values of recorded waveform samples instead of the amplitude. The sum, which is proportional to the amplitude, was calculated from high-pass filtered waveforms (to block any possible DC signal) over a sliding window of width $k+1/f_{0}$, which was moved by $1/f_{0}$ at a time. If the bubble echo started between $T_{i}$ and $T_{i+1}=T_{i}+1/f_{0}$, the whole echo (assuming it has the same shape as the reference signal) was in the sliding window starting at $T_{i}$ and the sum over the sliding window was significantly higher then in the case of no bubble (just noise). The threshold for detection of bubble presence was set in accordance with [25] as an average of noise plus 3–5 standard deviations. The transit time-dependent noise was measured before the experiment, with no bubbles in the measurement volume (only reflection from impurities and acoustic or electronic noise).

#### 2.2.2. Cross-Correlation Method

The cross-correlation is commonly applied to detect the delay of a signal with a known or assumed waveform [4,5,6,26,27,28,29,30]. A common error of the cross-correlation is the detection of a secondary maximum instead of the real one [26]. Unlike in [26], a normal pulse was applied and the error was dealt with by the tracking technique as described later. Also, in contrast with [28], no advanced filtering was applied, only a high-pass filter. The method was implemented as follows: After the thresholding detected a bubble echo starting in the interval $\left(T_{i},T_{i+1}\right)$, a small interval of the waveform (from $T_{i}$ to $T_{i+1}+k$) was cross-correlated with the reference signal for time lags ranging from 0 to $1/f_{0}$. The maximum value of the cross-correlation, the signal strength *M* (proportional to the amplitude of the bubble echo), was found and its corresponding lag $T^{\prime }$ was used to calculate the bubble echo transit time $T=T_{i}+T^{\prime }$. A limit for minimum transit time difference between two detected bubbles $T_{min}=\frac{3}{2}k$ ensured that each bubble was detected only once (the detection with higher *M* was chosen otherwise) in each pulse repetition’s waveform. Finally, all bubbles detected in the pulse were saved for the tracking as a point p=[t,T,M] including the measurement time *t* (the time when the pulse was emitted from the TDX), the transit time *T*, and the signal strength *M*.

The pulse-echo technique was implemented to analyse multiple pulse repetition waveforms in parallel. As a result of the parallel implementation and the fast combination of the thresholding and the cross-correlation, data could be processed online even with a mid-range processor (Intel Core i5-4690, Intel, Santa Clara, CA, USA). Moreover, the amount of data to save was reduced by 2–3 orders of magnitude to ~100 kB per second of the measurement, which could be handled by a regular hard drive and which makes it easy to conduct very long measurements.

### 2.3. Tracking Technique

**Figure 2:**
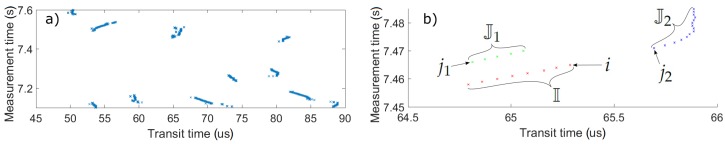
(**a**) Example of points for tracking. (**b**) The last point *i* of a trajectory I and the first point $j_{1}$ of a later trajectory $J_{1}$ were used to calculate the distance function $D(I,J_{1})$. The same way was used for trajectory $J_{2}$.

The task of the tracking technique was to connect detected bubble points (shown as points on the T−t plane in Figure 2a) to trajectories. The task was similar to clustering [31], which can either connect points to sets (agglomerative clustering) or divide the set of all points into smaller sets (divisive clustering). Our algorithm first used the measurement time *t* and transit time *T* (corresponding to the position) to connect points to trajectories and then divided some of these trajectories using the signal strength *M*. Unlike in [31], points in each trajectory were sorted by their measurement times, which allowed for improving the tracking performance (both its speed and fidelity).

#### 2.3.1. Connecting Points to Trajectories

First, all the points were considered trajectories with one point each. The tracking algorithm connected smaller trajectories to longer trajectories in cycles. The algorithm chose the most probable connection in each cycle and the cycle continued until there were no allowed connections left. The probability of connections was evaluated using a distance function D(I,J) (the smaller its value, the more probable the connection of trajectories I and J) and the distance function limit *l* decided which connections are not allowed. The algorithm continued until min(D(I,J))≥l.

The distance function was defined after considering the following. Since coalescence and break-up of bubbles were not considered, the trajectories should form one line without forking or going back in time. Furthermore, trajectories of two different bubbles should not be connected, even if they were positioned close to each other. Both requirements were achieved by not allowing any connection between trajectories with any overlap in the measurement time. As a consequence, the connection happens between the last point *i* of the preceding trajectory I and the first point *j* of the later trajectory J (Figure 2b) and other points do not have to be included (fast computation). The distance function was defined as:

(6) $$D\left(I,J\right)=\left\{\begin{array}{cc} \left(t_{j}-t_{i}\right)+ & \;\;w\left|T_{j}-T_{i}\right|\\ & \;\;\text{- for}\left\{0<\left(t_{j}-t_{i}\right)<t_{max}\right.\\ & \;\left.\wedge \;\;\;|T_{j}-T_{i}|\leq T_{max}\right\}\\ l & \;\;\text{- otherwise} \end{array}\right.$$

where $t_{max}$ and $T_{max}$ are respectively the measurement and transit time difference limits, and *w* is a scaling factor. The condition $0<(t_{j}-t_{i})$ represents the no-overlap condition. The measurement time limit $t_{max}$ was set higher than typical measurement time differences between points of one bubble and lower than typical measurement time differences between multiple bubbles or bubbles and noise points. In our case of the horizontal position measurement, the expected average velocity in the axial direction was 0. The transit time difference limit $T_{max}$ represents the highest allowed velocity ${\left|v\right|}_{max}=cT_{max}/2\left(t_{j}-t_{i}\right)$. The factor *w* was set as $w<t_{min}/T_{max}$, where the minimum allowed measurement time difference $t_{min}=1/f_{PRF}$ was the time difference between two consecutive pulses. This setting prioritised the measurement time differences over transit time differences and as a result, connections were made in the proper order and even trajectory points with slightly different transit times were not left out of the trajectory.

A trajectory of *N* connected points was treated as a N×3 array I=[t,T,M]. Because of the presence of noise points, the difference between noise and real trajectories was examined. In general, noise points were weak and sparse and thus could not connect to long trajectories due to $t_{max}$ and $T_{max}$ limits. A minimum signal strength $M_{min}$ and a minimum trajectory length $N_{min}$ were used to identify the noise. A trajectory was deleted if

(7) $$\max\left(M\right)<M_{min}\;\;\;\;\vee \;\;\;\;N<N_{min}.$$

#### 2.3.2. Dividing Trajectories Using the Signal Strength

**Figure 3:**
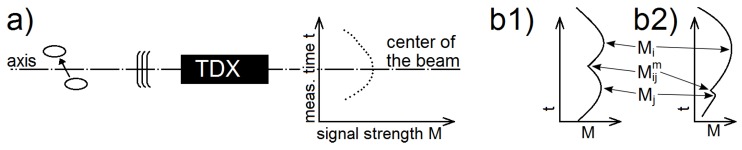
The signal strength was used to divide trajectories: (**a**) The typical signal strength of a bubble passing the beam axis. (**b1**) The signal strength for two connected bubbles had two clear peaks. (**b2**) The signal strength for one bubble with a noise. TDX: ultrasonic transducer.

The signal strength M of a bubble reflection is the largest when it is in the middle of the sound beam. A typical bubble has a signal strength, which first grows with the measurement time as the bubble is approaching the beam axis and then decreases as the bubble moves further away from the beam axis (Figure 3a). This behaviour was exploited for detecting multiple bubbles with similar transit times, which were incorrectly tracked as a single trajectory by the algorithm from Section 2.3.1.

The M−t signal of each connected trajectory was analysed to detect and divide those with multiple peaks (and thus multiple bubbles). Typical peak detection algorithms employ slope and amplitude thresholds to distinguish real peaks from noise. However, such an approach is not suitable in this case since both slope and amplitude can vary greatly between various bubbles due to differences in their vertical velocity, diameter, and horizontal distance from the beam axis. We applied a simple comparison of signal strengths at local maxima ($M_{i}$ and $M_{j}$) and at the minimum between them $M_{ij}^{m}$ (Figure 3b). The trajectory was divided at the minimum point of $M_{ij}^{m}$ when following conditions were met (thresholds 3 and 2 were determined empirically considering the signal condition during the experiment; the signal strength condition (7) was partially considered by the final condition):

(8) $$\begin{array}{cc} \max(M_{i},M_{j}) & >3\;M_{ij}^{m}\\ \min(M_{i},M_{j}) & >2\;M_{ij}^{m}\\ \min(M_{i},M_{j}) & >M_{min}. \end{array}$$

#### 2.3.3. Treating the Secondary Peak Detection Error of the Cross-Correlation Method

Because of noise, the shape of a reflection signal could change slightly. This change sometimes led to an error in the transit time estimation. Because the reflection signal and the reference signal were both nearly periodic with the basic frequency $f_{0}$, their cross-correlation was periodic as well and its maxima were spaced with $1/f_{0}$ between them. A common error of detecting the secondary maximum instead of the real one [26] resulted in transit time measurement error, which was a multiple of $1/f_{0}$. Since the axial velocity did not change much, it was possible to detect and correct this error by analysing all the points of a trajectory.

Because horizontal position was measured in this work, the average axial velocity was zero (bubbles travelled mostly vertically). Instantaneous velocities varied but stayed small, which was confirmed from the measured trajectories (besides occasional jumps to much higher values due to the error mentioned above). Assuming the real velocity was smaller than $\lambda f_{PRF}/4$, the transit time difference between two consecutive points in a trajectory should be smaller than $1/(2f_{0})$; therefore

(9) $$c_{i}-c_{i-1}=\left\lfloor \left(T_{i}-T_{i-1}\right)f_{0}+0.5\right\rfloor \;\;\;\;$$

should be zero. The $c_{i}$ is an index used for the detection of the discussed error. If the index difference was not zero, one of the two points was affected by the error. The next step was to decide which points are the correct ones and which are affected by the error. If only few points of a trajectory are affected by the error, most of the points have the same value of the coefficient $c_{i}$. Therefore, the value

(10) $$c^{correct}=\mathrm{mode}\left(c_{i}\right)\;\;\;\;$$

is most probably the correct one. Points with different coefficients were affected by the error and should be corrected as

(11) $$T_{i}^{new}=T_{i}+\frac{c^{correct}-c_{i}}{f_{0}}.$$

### 2.4. Experiment

**Figure 4:**
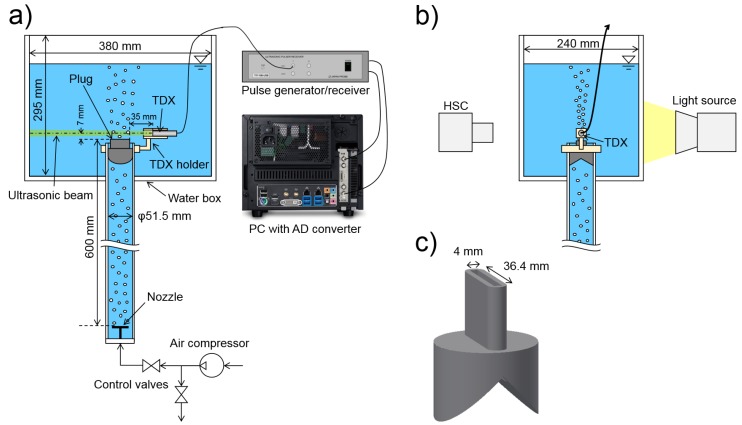
Experimental apparatus: (**a**) The front view. (**b**) The side view. (**c**) The detail of the plug and its narrow outlet channel. HSC: high-speed camera. AD: analog-to-digital.

The performance of the presented technique was evaluated experimentally using a reference concurrent measurement by an HSC. The experimental apparatus showed in Figure 4 was used for this purpose. Air was supplied by an air compressor and a pair of valves regulated the air flow rate. Air entered an acrylic pipe full of water through a nozzle with multiple holes and rose through the pipe in the form of bubbles. At the end of the pipe, there was a plug with a narrow inlet channel, through which bubbles entered an acrylic box filled with water.

Ultrasonic pulses were generated by the pulse generator/receiver (TIT-10B, JapanProbe, Yokohama, Japan) and repeated with frequency $f_{PRF}=1$ kHz. Each pulse was generated by two periods of basic frequency $f_{0}=1.8$ MHz. The TDX (1634 0102, Imasonic SAS, Besançon, France) with a 10-mm active diameter was fixed above the inlet. Received pulse waves went through a high-pass filter in the pulse generator/receiver and were digitised by the analog-to-digital (AD) converter (APX-5040, Aval Data, Tokyo, Japan) with a sampling speed of 150 MS/s. According to [32], the beam diameter was roughly estimated as D= 13–17 mm. The wavelength was λ= 0.83 mm and with that, constraints (2) and (5) were met.

#### 2.4.1. Calibration of the Pulse-Echo

It was explained that the position *x* in Equation (1) corresponds to the bubble vertex. However, reflections do not have a clear starting point nor does the reference signal. Furthermore, Equation (1) does not include the time delay required for an ultrasonic pulse to propagate through the TDX itself. Lastly, the speed of sound *c* changes not only due to temperature (which can be accounted for) but also due to impurities in the water. These issues are usually ignored due to their small effect. However, the current technique allowed achieving such a high level of accuracy that these issues became relevant. A calibration was performed to achieve as high measurement accuracy as possible.

Transit times were measured as relative to a TDX holder surface $x_{0}$. The transit time $T_{0}$ corresponding to $x_{0}$ and the precise speed of sound *c* were obtained by measuring reflections from an acrylic plate and analysing their transit times using the cross-correlation method with the same reference signal as for bubbles (only opposite since bubbles reflect with opposite phase compared to the acrylic plate). First, the acrylic plate was placed on the TDX holder surface (around 20 mm from the TDX) and the transit time $T_{0}$ was measured. Next, a hollow steel prism of a known length (39.95±0.05 mm) was used to place the acrylic plate to a well-known distance. The ultrasound travelled through the hole in the prism undisturbed and the corresponding transit time $T_{1}$ was measured. The speed of sound was evaluated using the difference of those transit times as

(12) $$c=\frac{2\times 39.95}{T_{1}-T_{0}}\pm \frac{2\times 0.05}{T_{1}-T_{0}}(\mathrm{mm}/s).$$

Finally, the position *x* of each reflection was obtained from the corrected transit time *T* as

(13) $$x=x_{0}+\frac{(T^{new}-T_{0})c}{2}\;\;\;.$$

#### 2.4.2. Reference Measurement with HSC

An HSC (Fastcam SA5, Photron, Tokyo, Japan) was used as a reference measurement technique to assess the pulse-echo performance. The HSC was positioned in front of the water box and was mounted with a Micro (Nikon, Tokyo, Japan) 60 mm lens (aperture f/4, depth of field 3 mm). The video was recorded at 1000 fps with the shutter speed of 1/20,000 s and the resolution of 1024 × 320 px. The backlight imaging method was used; the test section was illuminated from the rear by a metal-halide light (HVC-SL, Photron, Tokyo, Japan) and bubbles in the test section cast a shadow, which was recorded by the HSC.

The HSC footage was analysed using Matlab. The grey-scale threshold was applied to obtain a binary image from each video frame, which was then filtered using a morphological opening and closing. Bubble boundaries were detected at each frame using the Moore neighbour tracing algorithm with Jacob’s stopping criteria [33]. Bubbles were tracked through consecutive frames using a PTV algorithm similar to the one from [3]. The PTV algorithm used in this work considers only the change of the bubble centre position (calculated by averaging the positions of all points of the detected boundary). The two-dimensional (2D) position of the boundary point closest to the TDX was found in each frame, converted to mm using a reference length measurement (in both pixels and mm), and saved. The horizontal component of the position forms trajectories, which correspond to trajectories measured by the pulse-echo. The HSC video resolution was around 0.09 mm/px.

The plug with a 4 mm × 36.4 mm- wide channel (Figure 4c) directed all bubbles into a well-defined measurement volume. This helped to avoid bubbles overlapping on HSC since all were in roughly the same depth. The narrow measurement volume also ensured that all the bubbles crossed the ultrasound beam. As a consequence, all the bubbles were observed by the HSC clearly and the pulse-echo should have detected all bubbles as well. Therefore, trajectories can be directly compared for each bubble.

#### 2.4.3. Trajectory Association

In order to compare pulse-echo trajectories to HSC trajectories bubble-by-bubble, each trajectory was associated with its counterpart. A metric B(U,H) was used to define the similarity of a trajectory U (pulse-echo) with *N* points and a trajectory H (HSC) with *L* points. The metric was calculated as

(14) $$\begin{array}{cc} B(U, & \;H)=\left|t\left(U_{1}\right)-t\left(H_{1}\right)\right|+\left|t\left(U_{N}\right)-t\left(H_{L}\right)\right|+\left|<t{>}_{U}-<t{>}_{H}\right|\\ & \;+w^{\prime }\left(\left|x\left(U_{1}\right)-x\left(H_{1}\right)\right|+\left|x\left(U_{N}\right)-x\left(H_{L}\right)\right|+\left|<x{>}_{U}-<x{>}_{H}\right|\right) \end{array}$$

employing the time and the position from the start and the end of both trajectories, as well as trajectory averages. A scaling weight was set to $w^{\prime }=0.01$ s/mm, based on the 1 ms time resolution of both pulse-echo and HSC and the HSC video resolution of 0.09 mm. The metric was calculated for each possible pair of pulse-echo and HSC trajectories and trajectories were associated with their counterparts in an injective manner starting from the pair with the lowest metric.

## 3. Results

Bubble vertex trajectories were measured for nine different cases. Different air flow rates were applied in each case, which led to different bubble number densities (and void fractions). Out of 9 measurement cases, 6 cases were ~70 s long and 3 cases with high bubble number densities were ~30 s long; 250–750 bubbles were measured in each case. Bubbles were close to oblates in shape and their average horizontal and vertical lengths (around 3.9 × 2.1 mm based on HSC data) changed less than 2% between different measurement cases. The experimental condition is shown in Table 1.

**Figure 5:**
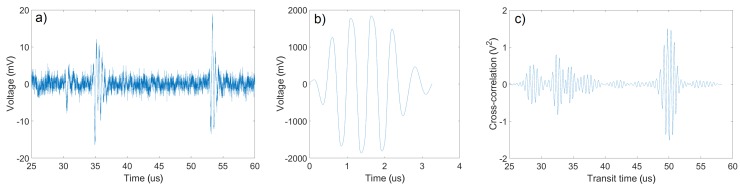
(**a**) An example of measured waveform (bubble reflections at T≈35μs and another at T≈53μs). (**b**) The reference signal. (**c**) An example of the cross-correlation function calculated from the waveform example.

**Figure 6:**
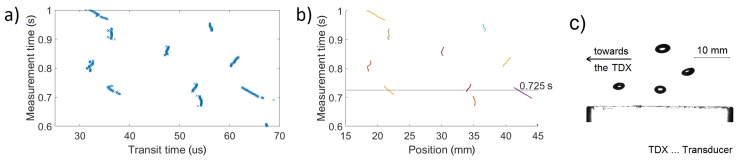
Example of results for case 9: (**a**) Points detected by pulse-echo. (**b**) Trajectories connected by the tracking technique. (**c**) HSC image for t=0.725 s. The top bubble has already left the sound field.

The 3.3-μs-long reference signal was measured as described in Section 2.2. An example of measured waveform is shown in Figure 5. Points were detected by the pulse-echo following the described procedure (an example is shown in Figure 6a). The tracking technique used the scaling factor w=200, the measurement time limit $t_{max}=4$ ms (4 repetitions), and the transit time difference limit $T_{max}=2.9$ μs (approximately 2.2 mm). The distance function limit l=10 ms was set higher than the maximum allowed distance function value. Therefore, it served only for identification of allowed and did not allow connections, as decided by $t_{max}$ and $T_{max}$. Values of the signal strength limit $M_{min}$ and the chain length limit $N_{min}$ were obtained from a stochastic analysis of detected trajectories. Both $M_{min}$ and $N_{min}$ were decreased for measurement cases with higher bubble number densities when the difference between bubbles and noise became less clear. Figure 6b shows the example of measured trajectories. A simultaneous detection of multiple bubbles can be confirmed for example for t=0.725 s in Figure 6c. This confirms our hypothesis that multiple bubbles can be measured simultaneously.

### Performance of the Technique

**Figure 7:**
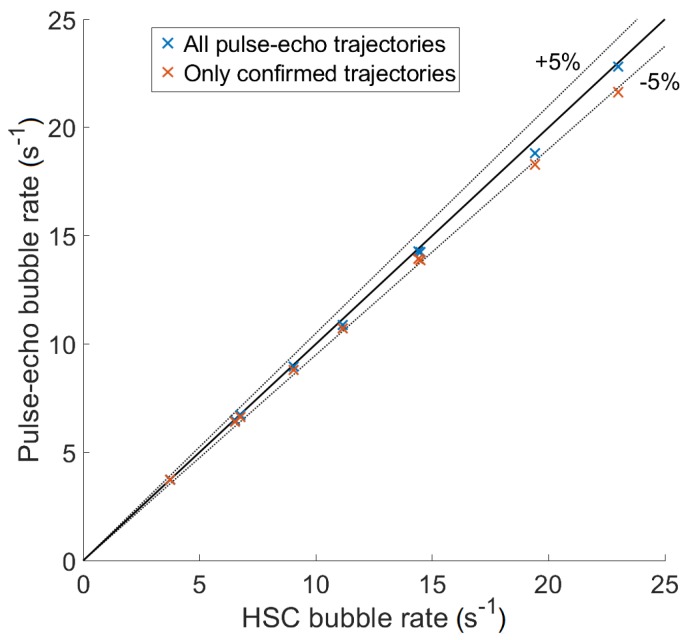
Comparison of the number of bubbles passing the measurement volume as measured by the pulse-echo with tracking and the HSC.

To confirm that the performance of the pulse-echo coupled with the tracking technique is reasonably good, results were compared with results of the HSC measurement. The number of trajectories (bubbles passing the measurement volume) was calculated for each case and divided by the total measurement time for both pulse-echo and HSC (Figure 7, Table 2). However, some of the pulse-echo trajectories might have been just a noise. It was assumed that HSC trajectories had no error and pulse-echo trajectories were associated with HSC ones (see Section 2.4.3). Because the pulse-echo failed to detect few bubbles, their HSC counterparts were left alone and were associated with noise pulse-echo trajectories. A noise like this was not too close to the HSC trajectory and could be identified. A measurement time interval $\left(t_{1},t_{2}\right)$ where both trajectories co-existed was detected. If the interval was longer than 1.5 ms (at least two points or frames) and the normalised position difference defined as

(15) $$x_{d}={\langle \left|x(U)-x(H)\right|\rangle }_{\left(t_{1},t_{2}\right)}$$

was smaller than 2 mm, the pulse-echo trajectory was confirmed to be correct; otherwise, it was a noise. Confirmed bubble rates (Figure 7, Table 2) show that the pulse-echo underestimates the bubble rate by around 5% for the highest bubble rates. This was due to two causes. First, some bubbles were not detected due to the combination of ultrasound field being blocked by other bubbles and the selection of the threshold value (Section 2.2.1). Second, some trajectories corresponding to multiple bubbles were not identified and divided (Section 2.3.2). Therefore, the pulse-echo detected fewer bubbles than the HSC. The blocking of bubbles could be reduced by optimising the selection of TDX diameter and wavelength. In addition, the simple condition (8) for detecting multiple connected bubbles could be improved to enhance the performance. The current technique underestimates the bubble rate by up to 5% and this number grows with the bubble rate. Moreover, the difference between all trajectories and confirmed trajectories shows that the rate of noise trajectories grows from 0 to around 5% as well. It can be assumed that both error rates will continue increasing for higher bubble rates.

The bubble rate discussed previously is not a commonly used two-phase flow parameter. Instead, the bubble number density is. In order to obtain the bubble number density, bubbles lying in a given volume have to be counted at each instant and their number divided by the volume. In two horizontal dimensions, the narrow inlet channel restricted the bubble positions. The HSC data confirmed that bubble vertices stayed uniformly distributed in a 36.4-mm-long region, which was the length of the inlet channel. It was assumed that bubbles were similarly restricted by the channel width of 4 mm. The vertical dimension of the volume required additional consideration.

**Figure 8:**
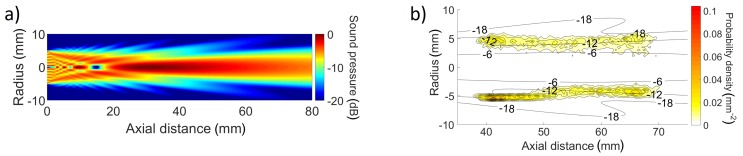
(**a**) Sound pressure field simulation based on [34]. (**b**) Detail with sound field in dB (contour lines) and the probability density of the position of the first or the last point of a pulse-echo trajectory.

For the pulse-echo, bubbles were detected only in the ultrasound field, which does not have a clear boundary (Figure 8a). The boundary was estimated using the HSC data. For each confirmed pulse-echo trajectory, the HSC provided the 2D location of the pulse-echo trajectory start and end. Figure 8b shows that the pulse-echo trajectories started and ended near the −12 dB level of sound pressure (compared with the maximum beam pressure). Therefore, the −12 dB level was considered to be the beam boundary. The area of sound pressure above −12 dB was integrated over the 36.4-mm-long axial distance interval to where bubbles were restricted. The surface was multiplied by the inlet channel width to obtain the volume of 1267 mm${}^{3}$. The instantaneous number of confirmed bubbles was calculated and then divided by the volume to get the bubble number density.

**Figure 9:**
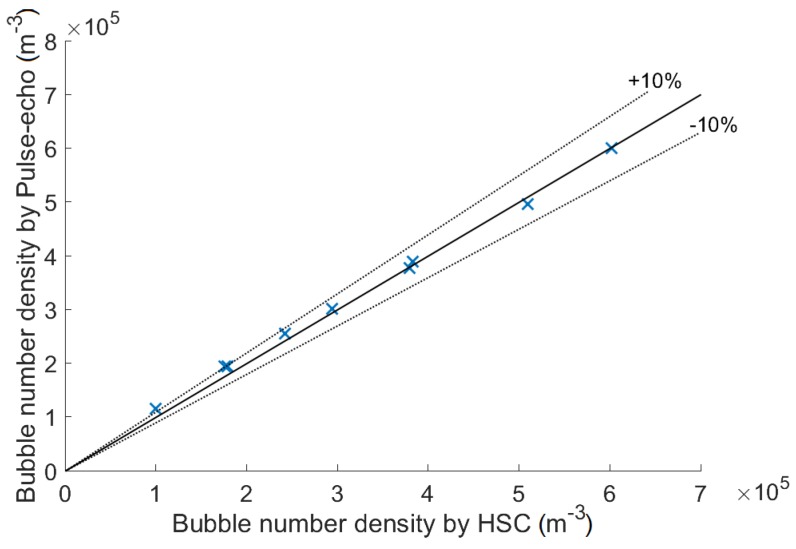
Bubble number densities by pulse-echo and HSC averaged over each measurement case.

The same volume was used for the HSC. The projection of the volume on the HSC image was 36.4-mm-long and its boundary followed the −12 dB sound pressure level. Bubbles with a vertex inside of this region were counted on each video frame and their count was divided by 1267 mm${}^{3}$ to get bubble number densities. Figure 9 and Table 3 show the comparison of bubble number densities averaged for each measurement case. Data agree well for high values with the pulse-echo overestimating low bubble number densities. This was caused by the uncertainty in determining the pulse-echo measurement volume, which seems to be actually larger than the −12 dB level. A larger volume would decrease the pulse-echo bubble number density and as a result, the comparison would be more similar to the comparison of bubble rates (Figure 7), which were calculated with fewer assumptions and uncertainties. However, the bubble number density data are more valuable as a reference. The maximum bubble number density ($0.6\times 10^{6}$ m${}^{-3}$) can be roughly compared to the void fraction of 1%, which was obtained by multiplying with the average bubble volume (Equation (10-30) in [35]) calculated using the average bubble axes 3.9 mm × 3.9 mm × 2.1 mm. As for the bubble rates, it can be assumed that error rates will steadily increase for higher void fractions and would still be reasonably low (depending on the condition, e.g., 20%) even for much higher void fractions. This is a subject of a future research along with the effect of void fraction oscillations caused by the nozzle bubble generator. The instantaneous bubble number densities reached as much as $3.2\times 10^{6}$ m${}^{-3}$ (void fraction 5.3%) and were 0 many times. More stable a bubble generator might result in similar error rates for higher volume fractions due to avoiding time intervals with void fraction 0% without increasing the volume fraction at moments when detection occurs.

## 4. Conclusions

The hypothesis that pulse-echo technique is capable of detecting multiple bubbles simultaneously has been confirmed. It can be seen from Figure 6b that at the time 0.725 s three bubbles were detected simultaneously. However, it has also been shown that the presence of multiple bubbles comes with a risk as the ultrasonic pulse gets distorted or completely blocked. Some of the measured trajectories were just noise and some bubbles were not detected by the pulse-echo. These problems, very rare for low bubble numbers, became more common as the bubble number increased. In the measurement case with the most bubbles (average bubble number density of $0.6\times 10^{6}$ m${}^{-3}$, void fraction of 1%), around 5% of detected trajectories were noise and about 5% of bubbles were not detected. Our results show that with a specific combination of TDX parameters, the error rates are reasonably small (<5%).

Nevertheless, the tested range was limited and the results can be quite different for different combinations of bubble diameter, wavelength, and TDX beam diameter. The simple consideration regarding these three parameters, which has been presented in this paper, could be improved upon, especially by analysing the sound diffraction behind bubbles and the reflection from bubble vertices positioned there in more detail. An improved understanding would allow for choosing more fitting a combination and thus improve the performance. Finally, it should be noted that the uncertainty in determining the pulse-echo measurement volume limits the application of this technique since bubble number density cannot be measured with high accuracy.

With that being said, the pulse-echo technique can resolve each bubble individually and by combining it with methods from [17,18,21] it can be possible to measure bubble diameters. These advantages have the potential to make the pulse-echo coupled with a tracking technique a valuable tool for studying bubbly flows with low bubble number densities/void fractions. This work, by overcoming the limitation of measuring only single bubbles, opens many possibilities for future applications and further development. The paper clearly explains the limitations of our work as well and thus it allows others to build on our results and to modify our technique as necessary.

## Figures and Tables

**Table 1:** Overview of the experimental condition.

Liquid	water
Gas	air
Morton number	2.2 × 10 ${}^{-11}$
Eotvos number	1.5
Weber number	2.1–2.8
Gas flow rate	up to 25 mL/min
Void fraction	up to 1%

**Table 2:** Comparison of the number of bubbles passing the measurement volume as measured by the pulse-echo with tracking and the HSC.

Bubble Rate (s${}^{-1}$)		
HSC	All Pulse-Echo	Confirmed
3.75	3.72	3.72
6.52	6.48	6.40
6.76	6.72	6.63
9.05	8.95	8.81
11.14	10.87	10.72
14.48	14.26	13.87
14.40	14.27	13.94
19.41	18.83	18.29
22.99	22.80	21.63

**Table 3:** Bubble number densities by pulse-echo and HSC averaged over each measurement case.

Bubble Number Density (m${}^{-3}$)	
HSC	Pulse-Echo
100,160	116,730
175,870	194,810
178,870	194,060
242,500	255,990
294,440	301,940
383,200	390,600
379,310	378,860
509,740	498,390
601,620	604,020

## Abbreviations

The following abbreviations are used in this manuscript:

CFD	Computational fluid dynamics
HSC	High-speed camera
PTV	Particle tracking velocimetry
TDX	Ultrasonic transducer
URRTT	Ultrasound reflector recognition and tracking technique

## Nomenclature

B(U,H) (s)	Metric between pulse-echo trajectory U and HSC trajectory H	$N_{min}$ (-)	Minimum trajectory length
		*t* (s)	Measurement time
*c* (m/s)	Speed of sound	$t_{min}$ (s)	Minimum measurement time difference
$c_{i}$ (-)	Category of point *i*		
$c^{correct}$ (-)	The most common category	$t_{max}$ (s)	Measurement time difference limit
*d* (m)	Bubble diameter	*T* (s)	Transit time
*D* (m)	Ultrasonic beam diameter	$T_{s}$ (s)	Waveform length
D(I,J) (s)	Distance function of trajectories I and J	$T^{\prime }$ (s)	Time lag of cross-correlation
$f_{0}$ (s${}^{-1}$)	Basic frequency	$T_{0}$ (s)	Reference transit time
$f_{PRF}$ (s${}^{-1}$)	Pulse repetition frequency	$T_{1}$ (s)	Transit time measured for calibration
H	HSC trajectory (L× 3 array)	$T_{min}$ (s)	Minimum transit time difference
*i*, *j*	Detected point (1 × 3 vector)	$T_{max}$ (s)	Transit time difference limit
I, J, U	Pulse-echo trajectory (N× 3 array)	$T^{new}$ (s)	Corrected transit time
*k* (s)	Reference signal length	*v* (m/s)	Bubble surface velocity
*l* (s)	Distance function limit	*w* (-)	Scaling factor
L,N (-)	Trajectory length (number of points)	$w^{\prime }$ (s/m)	Scaling factor
*M* (V${}^{2}$)	Signal strength of a reflecting point	*x* (m)	Position
$M_{min}$ (V${}^{2}$)	Signal strength limit	$x_{0}$ (m)	Reference position
$M_{ij}^{m}$ (V${}^{2}$)	Minimum signal strength	$x_{d}$ (m)	Normalised position difference
$M_{i},M_{j}$ (V${}^{2}$)	Local maximum in trajectory signal strength	λ (m)	Wavelength
*n* (-)	Period count per pulse		
